# Life dissatisfaction in Canadians aged 40 and above with cancer and mental health disorders: A cross‐sectional study using the Canadian Community Health Survey

**DOI:** 10.1002/cam4.4287

**Published:** 2021-09-28

**Authors:** Danielle Carole Roy, Ronda Lun, Tzu‐Fei Wang, Yue Chen, Philip Wells

**Affiliations:** ^1^ School of Epidemiology and Public Health Faculty of Medicine University of Ottawa Ottawa Ontario Canada; ^2^ Department of Medicine University of Ottawa, and The Ottawa Hospital Research Institute Ottawa Ontario Canada

**Keywords:** cancer, chronic disease, mental health, personal satisfaction, psycho‐oncology, quality of life

## Abstract

**Background:**

Life dissatisfaction varies with different factors––particularly in the presence of chronic conditions, such as cancer. The combination of cancer and mental health disorders may increase life dissatisfaction due to lowered resilience against stress. We sought to determine if life dissatisfaction is higher in Canadians aged 40 and above with cancer compared to the cancer‐free population and if there is a synergistic effect between cancer and mental health disorder on life dissatisfaction.

**Methods:**

We conducted a cross‐sectional study using the 2015–2016 Canadian Community Health Survey. We included 67,294 subjects aged 40+, and evaluated the association between cancer, mental health disorders, and life dissatisfaction using logistic regression and odds ratios (ORs) while adjusting for age, sex, marital status, education level, and chronic conditions. Relative excess risk due to interaction (RERI), attributional proportion due to interaction (AP), and Synergy index (S‐index), were calculated to determine the significance of additive interaction.

**Results:**

Compared to the cancer‐free population, life dissatisfaction was higher in patients with cancer (OR 2.44, 95% CI: 1.88–3.16) and mental health disorders (OR 5.17, 95% CI: 4.56–5.85). The adjusted ORs for life dissatisfaction were 2.45 (95% CI: 1.74–3.43) and 5.17 (95% CI: 4.55–5.87) for cancer and mental health disorders, respectively, but when both conditions were present, the OR increased to 12.50 (95% CI: 8.40–18.62). The results suggested a synergistic interaction (RERI: 5.89 [95% CI: 0.91–10.87]; AP: 0.47 [95% CI: 0.25–0.69]; and S‐index: 2.05 [95% CI: 1.30–3.23]).

**Conclusion:**

This study showed higher life dissatisfaction in cancer and mental health disorder patients. A synergistic effect was detected between cancer and mental health disorder on life dissatisfaction. These results suggest cancer patients with mental health disorders require additional support and psychological resources to improve their quality of life.

**Lay Summary**

Individuals with a chronic health condition, such as cancer, may have higher life dissatisfaction––an increasingly measured research outcome since improvement in personal well‐being has become a societal aspiration. This study showed higher life dissatisfaction in Canadians aged 40 and above with cancer and mental health disorders. A synergistic effect was detected between cancer and mental health disorder on life dissatisfaction. These results suggest cancer patients with mental health disorders require additional support and psychological resources to improve their quality of life.

## 1 INTRODUCTION

Life satisfaction is a key psychosocial aspect of subjective well‐being relevant to health and quality of life (QoL).^1^ It is conceptualized as a subjective assessment of the QoL and the degree to which it matches self‐imposed life standards.^2^ Life satisfaction has been shown to vary according to many factors, including marital status, age, sex, education, income, mental health, and chronic conditions.^3^, ^4^, ^5^, ^6^ According to the literature, poor life satisfaction (or life dissatisfaction) is especially recognized in individuals with cancer^7^ and mental health disorders (i.e., anxiety disorders and major depression).^8^, ^9^ Previous research showed increased life dissatisfaction in Spanish cancer patients compared to the non‐cancer population.^7^ Life dissatisfaction is suggested to be high in individuals with cancer due to the physical and psychological ramifications associated with cancer diagnosis and treatment (i.e., cognitive impairment, infertility, fatigue, and pending death) which can create important psychological distress and psychosocial impairment.^10^, ^11^, ^12^, ^13^ On the other hand, there is little research surrounding life dissatisfaction in Canadians living with cancer. Given life dissatisfaction is strongly influenced by a country's level of social capital,^14^ the results from Spain may not be applicable to Canada and country‐specific studies are needed. The anticipated increase in the incidence of cancer in Canada^15^, ^16^ further supports the need to quantify and understand the psychological well‐being of Canadians living with cancer.

Literature shows significantly higher life dissatisfaction in those with major depression, anxiety, and substance‐dependent disorders compared to those without.^9^ The state of one's mental health may also be exacerbated by the development of a chronic condition, such as cancer, and the associated stress.^17^ It is suggested that patients with mental health disorders diagnosed with a health condition may have lifestyle adjustment difficulties causing significant stress which can produce symptoms of malaise, irritability, and fatigue.^17^, ^18^, ^19^ These symptoms may have a significant impact on the QoL ultimately resulting in life dissatisfaction.

Henceforth, this research aims to assess the questions: is life dissatisfaction higher in Canadians aged 40+ with cancer compared to the cancer‐free population, and is there a synergistic effect between cancer and mental health disorders on the prevalence of life dissatisfaction? Given the physical and psychological impairments associated with cancer,^10^, ^11^ we hypothesize higher life dissatisfaction in Canadians with cancer and that there will be a synergistic effect between cancer and mental health disorders on life dissatisfaction.

## 2 METHODS

### 2.1 Study design

We conducted a cross‐sectional study utilizing data from the 2015–2016 Canadian Community Health Survey (CCHS) public use microdata file to inspect life dissatisfaction in Canadians with and without cancer, as well as to evaluate the possible synergistic effect between cancer and mental health disorders on life dissatisfaction in Canadians. The CCHS is a cross‐sectional survey that uses a multistage sampling strategy which is designed to collect information on the health status, health care utilization, and health determinants from a large sample of the Canadian population every 2 years to attain reliable estimates at the health region level.^20^ The use of a multistage sampling strategy serves to get a fair sample distribution of the health regions and provinces as it samples and collects population data in stages. For the conduct of this study, we followed the Strengthening of the Reporting of Observational Studies in Epidemiology (STROBE) reporting and the Reporting of Studies Conducted Using Observational Routinely Collected Health Data (RECORD) guidelines.^21^, ^22^

### 2.2 Study population

**FIGURE 1:**
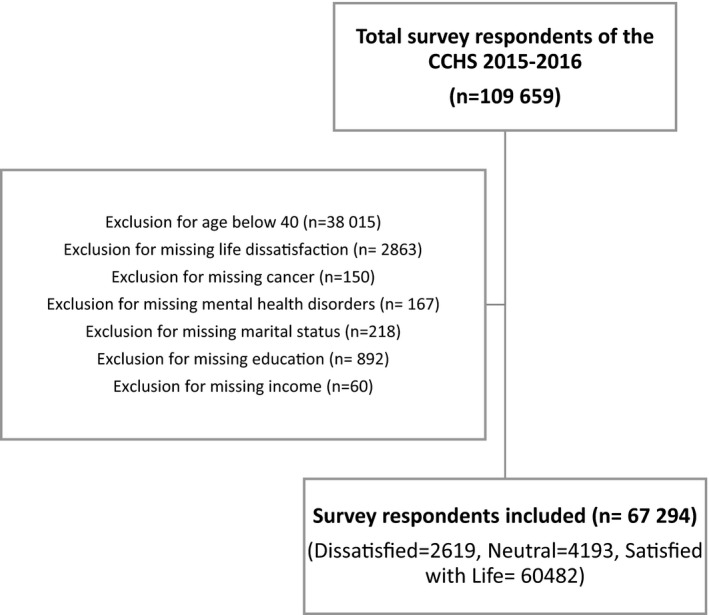
Flow diagram showing selection of study participants

The CCHS included people aged 12 years and older in all provinces and territories but excluded those from the Canadian Forces, living in First Nation Reserves/settlements and Crown Lands, youths aged 12–17 in foster homes, and certain individuals living in remote areas.^20^ For the purpose of this study, the population of interest was Canadian residents aged 40+. We excluded subjects under the age of 40 (*n* = 38,015) and those with missing information for any of the included variables (i.e., life dissatisfaction [*n* = 2863], cancer status [*n* = 150], mental health disorders [*n *= 167], and study covariates [*n* = 1170]) (Figure 1). Therefore, the sample population consisted of 67,294 Canadian residents aged 40+.

### 2.3 Variables

Life dissatisfaction, the primary outcome variable, was determined by the question ‘How satisfied are you with your life in general?’ with the self‐reported response options being: (1) very satisfied; (2) satisfied; (3) neither satisfied nor dissatisfied; (4) dissatisfied; and (5) very dissatisfied. Due to small cell sizes and strict interest in looking at dissatisfaction as the main event, the outcome was divided into two groups: Satisfied with life/neutral (0) and not satisfied with life (1). We chose to pool life satisfaction with ‘neither satisfied nor dissatisfied’ to include all people who were not dissatisfied with life as the reference group. Cancer and mental health disorders were dichotomized into the self‐reported presence or absence of cancer and having a self‐reported present mood/anxiety disorder in the CCHS, respectively. A subjects cancer status was determined by the question ‘Do you have cancer?’ (Yes/No) while their mental health status was decided by the questions ‘Do you have a mood disorder such as depression, bipolar disorder, mania or dysthymia?’ (Yes/No) and ‘Do you have anxiety disorder such as a phobia, obsessive‐compulsive disorder or a panic disorder?’ (Yes/No). The model was adjusted for covariates that are considered to be potential confounders according to the literature: age, sex, marital status, education level, mental disorders, and chronic conditions.^3^, ^4^, ^5^, ^6^ Age was categorized into five categories of 10‐year intervals: 40–49, 50–59, 60–69, 70–79, and 80 or over. Annual household income was recategorized in tertiles around the median income of Canadian families.^23^ Marital status was categorized into four categories: married, common‐law, widowed/separated, and single. Education level of the individual was categorized into three categories: less than secondary school graduation, secondary school graduation but no post‐secondary education, and post‐secondary education. Sex and presence of a chronic condition were categorized as dichotomous variables with ‘chronic condition’ denoting having one of the self‐reported chronic conditions in the CCHS other than cancer. The included chronic conditions consists of joint pain, asthma, chronic obstructive pulmonary disorder, sleep apnea, scoliosis, fibromyalgia, arthritis, back problems, osteoporosis, high blood pressure, hypercholesteremia, heart disease, diabetes, and migraine headaches.

### 2.4 Statistical analysis

Given the complex survey design of the CCHS, average sampling weight and design effect were taken into consideration to produce estimates specific to the Canadian population. Since the public use data files of the CCHS does not include information on the details of the survey design, we used an approximate approach incorporating sample weight and design effect to calculate adjusted weights using the method previously described by Chen et al.^24^ To compute descriptive statistics, we used frequency tables between study variables and life dissatisfaction. We used unweighted results to attain the number of respondents in each group, meanwhile we used weighted results for the prevalence and inferential analysis. To assess the association between life dissatisfaction and the covariates, we used the Cochran‐Mantel‐Haenszel statistic. A *p value* below 0.05 suggested a statistically significant association.

To assess the association between cancer and life dissatisfaction, we used a multivariable weighted logistic regression model before and after controlling for important covariates. The proc logistic SAS procedure was utilized to fit the weighted models. Model parameters were estimated using maximum likelihood estimation and the significance of each adjusted covariate was tested using the Wald test. Effect modification on a multiplicative scale was assessed in the model using a stepwise forward approach, which consisted of adding one interaction term at a time and investigating the significance of the interaction. A statistically significant interaction was based on *p* value <0.1. When a significant interaction was found, we performed a likelihood‐ratio test to compare the fit between models to determine whether the interaction term should be included. A *p* value below 0.1 suggested that the new model offers significantly better goodness of fit than the original model resulting in the inclusion of the interaction term.

To assess the additive interaction between cancer and mental health disorder, we again used a multivariable weighted logistic regression model achieved using the proc logistic SAS procedure, to obtain odds ratios (ORs) and 95% confidence intervals (CIs) for the combination of two exposures. For the purpose of this analysis, the variables, cancer status, and mental health disorder were combined allowing us to assess the independent and combined effect of cancer status and mental health disorder on life dissatisfaction. To determine whether additive interaction was present, we calculated the relative excess risk due to interaction (RERI), the attributional proportion due to interaction (AP), and Synergy/Rothman's index from the adjusted ORs using the formulated Excel sheet created by Andersson et al.,^25^ which is set up to calculate the measures of additive interaction listed precedingly. These measures are used to quantify the interaction on an additive scale.^26^ A statistically significant additive interaction was based on whether the RERI and AP were statistically different than 0 and the Synergy index being statistically different than 1.

A sensitivity analysis was conducted to determine the influence of missing data by regrouping those with missing cancer into the ‘No cancer’ category, those with missing mental health disorder into the ‘No mental health disorder’, and for other covariates, those with missing information were grouped into their reference groups. Logistic regression models were refitted. All statistical analyses, other than the RERI, AP, and S‐index calculations, were performed in SAS version 9.4 (SAS Institute, Inc.).

## 3 RESULTS

The prevalence of life dissatisfaction was 5.45% and 12.12% in individuals with cancer and mental health disorders, respectively. Approximately a quarter of individuals with cancer and mental health disorder (28.41%) reported life dissatisfaction compared with only 1.98% for cancer‐free individuals with no self‐reported mental health disorder (Table 1). There were statistically significant associations of life dissatisfaction with cancer, mental health disorder, and the combination of cancer and mental health disorder. (Table 1; Table S1).

**TABLE 1:** Prevalence of life dissatisfaction of Canadian adults according to cancer and mental health disorder: Canadian Community Health Survey 2015–2016

Mental health disorder	Cancer	No.	Dissatisfied with life	%^a^	*p* value
No	No	57,073	1281	1.98	<0.0001
No	Yes	1487	81	5.45	
Yes	No	8455	1189	12.12	
Yes	Yes	279	68	28.41	

^a^ Weighted to the Canadian population.

Table 2 shows the results from the logistic regression analysis. The adjusted model included cancer, mental health disorders, and covariates (age, sex, education level, income, chronic condition, and marital status). All covariates but sex was associated with the outcome in the general population (Table S1). There was no significant interaction between cancer and other covariables. The OR for life dissatisfaction was 2.44 (95% CI: 1.88–3.16) in individuals with cancer compared to cancer‐free individuals and was 5.17 (95% CI: 4.56–5.85) in those with mental health disorders compared to individuals without mental health disorders.

**TABLE 2:** Odds ratios (ORs) and 95% confidence intervals (CIs) for life dissatisfaction associated with cancer, mental health disorder, and covariates: Logistic regression analysis

Risk factors		Crude		Adjusted	
		OR (95% CI)	*p* value	OR (95% CI)	*p* value
Cancer	No	1.00 (reference)	<0.0001	1.00 (reference)	<0.0001
	Yes	3.00 (2.32–3.87)		2.44 (1.88–3.16)	
Sex	Female	1.00 (reference)	0.3857	1.00 (reference)	<0.0001
	Male	1.05 (0.94–1.18)		1.46 (1.30–1.65)	
Mental health disorder	No	1.00 (reference)	<0.0001	1.00 (reference)	<0.0001
	Yes	6.81 (6.04–7.69)		5.17 (4.56–5.85)	
Education level	Less than secondary school graduation	1.00 (reference)		1.00 (reference)	
	Secondary school graduation	0.63 (0.54–0.75)	<0.0001	0.81 (0.68–0.96)	0.0136
	Post‐secondary education	0.43 (0.37–0.49)	<0.0001	0.66 (0.56–0.77)	<0.0001
Income	<$40,000	1.00 (reference)		1.00 (reference)	
	$40,000–79,999	0.42 (0.36–0.48)	<0.0001	0.59 (0.51–0.68)	<0.0001
	$80,000+	0.19 (0.16–0.22)	<0.0001	0.34 (0.29–0.41)	<0.0001
Chronic condition	No	1.00 (reference)	<0.0001	1.00 (reference)	<0.0001
	Yes	3.29 (2.67–4.06)		2.24 (1.80–2.78)	
Age (years)	40–49	1.00 (reference)		1.00 (reference)	
	50–59	1.31 (1.12–1.54)	0.0007	1.12 (0.95–1.31)	0.1674
	60–69	1.23 (1.04–1.45)	0.0170	0.98 (0.82–1.16)	0.7861
	70–79	1.16 (0.94–1.42)	0.1659	0.82 (0.66–1.02)	0.0702
	80+	1.71 (1.33–2.21)	<0.0001	1.01 (0.77–1.32)	0.9420
Marital status	Single	1.00 (reference)		1.00 (reference)	
	Divorced/separated/widowed	0.81 (0.69–0.95)	0.0096	0.86 (0.72–1.02)	0.0730
	Common‐law	0.27 (0.21–0.35)	<0.0001	0.43 (0.33–0.56)	<0.0001
	Married	0.26 (0.22–0.30)	<0.0001	0.49 (0.41–0.57)	<0.0001

Table 3 presents the crude and adjusted OR of the independent and joint effect of cancer and mental health disorders on life dissatisfaction. After adjustment for sex, age, education level, income, marital status, and chronic conditions, the OR was 2.45 (95% CI: 1.74–3.43) for the independent effect of cancer and was 5.17 (95% CI: 4.55–5.87) for the independent effect of mental health disorders. The OR for the joint effect of cancer and mental health disorder on life dissatisfaction was 12.50 (95% CI: 8.40–18.62). The RERI was 5.89 (95% CI: 0.91–10.87), the AP was 0.47 (95% CI: 0.25–0.69), and S index was 2.05 (95% CI: 1.30–3.23). The results from sensitivity analysis showed no notable changes (see Tables S2 and S3).

**TABLE 3:** Unadjusted and adjusted measures of association between cancer status and self‐reported mental health disorder on the prevalence of life dissatisfaction using logistic model^a^ for OR

Mental health disorder	Cancer	Unadjusted OR (95% CI)	Adjusted^b^ OR (95% CI)
No	No	1.00 (reference)	1.00 (reference)
No	Yes	2.73 (1.91–3.89)	2.45 (1.74–3.43)
Yes	No	6.74 (5.95–7.64)	5.17 (4.55–5.87)
Yes	Yes	18.55 (12.45–27.64)	12.50 (8.40–18.62)

^a^ Weighted to the Canadian population.

^b^ Adjusted for sex, age, education level, income, marital status, and chronic conditions.

## 4 DISCUSSION

To the best of our knowledge, this is the first study assessing the association between life dissatisfaction and cancer as well as the potential interaction between cancer and mental health disorders on life dissatisfaction in Canadians aged 40+. Our results suggest that the odds of life dissatisfaction are higher in subjects with cancer, even after adjusting for sex, age, mental health disorders, income, marital status, education, and chronic conditions. Additionally, we found that the prevalence and adjusted OR of life dissatisfaction were the highest in individuals with both cancer and mental health disorders (12.50 vs. 2.45 and 5.17 for combined vs. cancer vs. mental health disorders). According to the measures of additive interaction, a significant synergistic effect was discovered.

Similar findings have been seen in previous studies from other countries in regards to higher life dissatisfaction in cancer patients. Particularly, in the study of Vázquez et al.,^7^ the authors found that people from Spain with cancer had significant reductions in levels of life satisfaction compared to the cancer‐free population. Interestingly, the authors noted cancer as having the highest impact on life dissatisfaction when compared to other health conditions including diabetes, osteoporosis, etc.^7^ The current literature suggests that life dissatisfaction in cancer patients is due to reduced QoL from cancer‐related fatigue, inability to work, worry, anxiety, stress, and bodily changes such as climacteric and other hormonal problems.^27^, ^28^ It has also been reported that some experience reduced social networks during the disease process causing reduced QoL and higher perceived stress.^29^ However, some studies also report that not all cancer patients suffer from life dissatisfaction. It is suggested that those with effective coping skills, social support, and engagement in personally meaningful activities tended to report lower levels of life dissatisfaction.^30^, ^31^ Thus, offering sources of social support and development of coping skills may enable patients to effectively manage psychological distress or negative symptoms, translating to decreased levels of life dissatisfaction. Previously, Dubey and Agarwal^32^ have reported the use of combined active coping and humor coping strategies as predictors of satisfaction with life in cancer patients. While we could not evaluate the prevalence of life dissatisfaction in relationship to different cancer types and treatments since this information is not provided in the CCHS, previous studies had indicated that these factors could impact the level of life dissatisfaction. For instance, breast cancer has been found to be attributed with the lowest QoL in the psychological domain compared to other cancer types, with prostate cancer having the highest QoL in the psychological domain.^33^ The use of alternative medicine has also been associated with greater psychosocial distress and worse quality of life.^34^ Future studies should aim to investigate the differences of life dissatisfaction among patients undergoing different therapies and with different cancer types.

The finding of positive additive interaction is consistent with a previous study which concluded that there is an additive effect of comorbid physical and/or psychological problems on life dissatisfaction.^7^ Patients with a chronic comorbid condition have to adjust their lifestyle, aspirations, and employment––processes that may be difficult to do in patients with mental health disorders without triggering significant stress.^17^, ^18^ Since this study is cross‐sectional, the orders of the occurrence of conditions were unknown. It is probable that patients develop a psychiatric disorder, commonly depression or anxiety, as a result of their chronic condition.^17^ This would argue for interventions early in the illness experience to improve psychological distress and subjective well‐being.^35^ On the other hand, chronic stress can be a risk factor for chronic health conditions.^36^ Therefore, further research on the relationship is needed to understand the pathophysiology and interactions between stress and disease.

This study has important clinical implications. The results suggest the need to implement psychosocial interventions, simultaneous to cancer treatments, particularly in those who have mental health disorders. There are studies reporting improvement in cancer patients’ psychological well‐being and/or QoL following psychological interventions such as coping strategies (i.e., combined active coping, humor coping, positive religious coping, hope, and spirituality)^32^, ^37^, ^38^, ^39^ and cognitive‐behavioral stress management.^40^, ^41^ Interestingly, existing research does suggest that good psychological well‐being (i.e., low life dissatisfaction) may have a causal effect on some parameters of health, including recovery.^42^, ^43^, ^44^ From the studies of Chang et al.^45^ and Lee et al.,^46^ this may be especially important in those with both cancer and mental health disorders since improving one's mental health may lead to improved cancer prognosis and survival. This highlights the importance of focusing on not only illness and disability, but also an individual's psychological well‐being and resilience.^1^, ^47^ The results support the development of public policies aimed at optimizing the subjective well‐being, QoL, and diminishing life dissatisfaction in cancer patients for the improvement of disease.

Although we were able to capture the prevalence of life dissatisfaction in Canadians living with cancer and mental health disorders, this study has limitations. First, due to the self‐reported nature of the cancer and mental health disorder variables, they are vulnerable to misclassification and errors from non‐response, reporting, and recall bias. Specifically, respondents may differ from non‐respondents and mental health disorders and cancer may be underreported which might have resulted in an underestimated association. It is also possible that patients who reported having cancer or a mental health disorder were more likely to report life dissatisfaction which might have resulted in an overestimation of the results. Thus, future studies should aim on using objective, non‐self‐reported data (i.e., administrative data). In addition, as life dissatisfaction may differ by mental health disorders, evaluation by the type of mental disorder could also be of interest. Additionally, with a high mortality condition such as cancer, respondents were more likely to have a milder form of the disease, thereby possibly excluding those with severe or terminal cancer. Second, cancer as well as the combined group of cancer and mental health disorders represented small proportions of our study population (2.20% and 0.36%, respectively). Third, since we excluded patients with missing data, there may be selection bias since they may have been different compared to the individuals analyzed. Fourth, given the cross‐sectional nature of this study, we cannot infer a causal relationship between cancer and life dissatisfaction. In fact, a meta‐analysis showed that psychological well‐being including life dissatisfaction may have a causal effect on objective health outcomes.^43^ Despite these limitations, the strengths of the study included the large sample size from CCHS, covering ~98% of the Canadian population and the analysis approach which integrated methods for weighting and substantial covariates adjustment.

## 5 CONCLUSION

Canadians with cancer are more likely to suffer life dissatisfaction compared to those who are cancer‐free. Our findings also showed a significant synergistic effect between cancer and mental health disorders on the prevalence of life dissatisfaction. The results highlight the importance of addressing life dissatisfaction and the need to consider implementing and utilizing psychosocial interventions simultaneous to cancer treatments for QoL improvement in the cancer population.

## CONFLICT OF INTEREST

There is no conflict of interest.

## ETHICS APPROVAL

No ethics approval was necessary for this study because it uses publicly accessible data.

## Supporting information

Table S1‐S3

## Data Availability

The data used for this study––the 2015–2016 CCHS microdata file––are available for public use via Statistics Canada (https://www150.statcan.gc.ca/n1/en/catalogue/82M0013X2019001). The dataset creation plan and analytics codes utilized for the purpose of this study are available from the authors of this manuscript upon request.
