# Supplementary material for: Life dissatisfaction in Canadians aged 40 and above with cancer and mental health disorders: A cross‐sectional study using the Canadian Community Health Survey
Source: Cancer Med. 2021 Sep 28;10(21):7601–9. doi: 10.1002/cam4.4287 (PMC8559453; doi:10.1002/cam4.4287)
Supplement: Supplementary file 1 — Table S1‐S3 [file CAM4-10-7601-s001.docx]

**SUPPLEMENTARY MATERIALS**

**SM Table 1.** Prevalence of life dissatisfaction of Canadian adults according to Covariates: Canadian Community Health Survey 2015-2016

| **Risk factors** | **No.** | **Dissatisfied with life** | **%^a^** | ***P-value*** |
| --- | --- | --- | --- | --- |
| **Mental health disorder** |  |  |  | *<0.0001* |
| No | 58560 | 1362 | 2.06 |  |
| Yes | 8734 | 1257 | 12.63 |  |
| **Cancer** |  |  |  | *<0.0001* |
| No | 65528 | 2470 | 3.15 |  |
| Yes | 1766 | 149 | 9.19 |  |
| **Age (years)** |  |  |  | *0.0009* |
| 40-49 | 13921 | 492 | 2.75 |  |
| 50-59 | 17553 | 853 | 3.54 |  |
| 60-69 | 18607 | 682 | 3.36 |  |
| 70-79 | 11642 | 392 | 3.18 |  |
| 80+ | 5571 | 200 | 4.64 |  |
| **Sex** |  |  |  | *0.3596* |
| Male | 30576 | 1204 | 3.20 |  |
| Female | 36718 | 1415 | 3.37 |  |
| **Marital status** |  |  |  | *<0.0001* |
| Married | 33788 | 731 | 2.03 |  |
| Common-law | 5456 | 129 | 1.99 |  |
| Widowed or  Separated or divorced | 19310 | 1133 | 5.89 |  |
| Single | 8740 | 626 | 7.25 |  |
| **Education level** |  |  |  | *<0.0001* |
| Less than secondary school graduation | 12509 | 746 | 5.80 |  |
| Secondary school graduation | 14941 | 621 | 3.75 |  |
| Post-secondary education | 39844 | 1252 | 2.56 |  |
| **Annual household income** |  |  |  | *<0.0001* |
| <$40,000 | 21153 | 1582 | 7.27 |  |
| $40,000 – $79,999 | 20970 | 624 | 3.10 |  |
| $80,000+ | 25171 | 413 | 1.45 |  |
| **Chronic condition** |  |  |  | *<0.0001* |
| No | 11644 | 145 | 1.19 |  |
| Yes | 55650 | 2474 | 3.83 |  |

*^a^ Weighted to the Canadian population*

**SM Table 2.** Sensitivity analysis* using odds ratios (ORs) and 95% confidence intervals (CIs) for life dissatisfaction associated with cancer, mental health disorder and covariates: Logistic regression analysis

| **Risk factors** | | **Adjusted** | |
| --- | --- | --- | --- |
|  |  | **OR (95% CI)** | **P-value** |
| **Cancer** | No | 1.00 (reference) | *<0.0001* |
|  | Yes | 2.58 (2.01-3.32) |  |
| **Sex** | Female | 1.00 (reference) | *<0.0001* |
|  | Male | 1.42 (1.26-1.60) |  |
| **Mental health disorder** | No | 1.00 (reference) | *<0.0001* |
|  | Yes | 4.99 (4.42-5.63) |  |
| **Education level** | Less than secondary school graduation | 1.00 (reference) |  |
|  | Secondary school graduation | 0.78 (0.66-0.91) | *0.0023* |
|  | Post-secondary education | 0.63 (0.54-0.72) | *<0.0001* |
| **Income** | <$40,000 | 1.00 (reference) |  |
|  | $40,000 – 79,999 | 0.63 (0.55-0.72) | *<0.0001* |
|  | $80,000+ | 0.37 (0.31-0.43) | *<0.0001* |
| **Chronic condition** | No | 1.00 (reference) | *<0.0001* |
|  | Yes | 2.29 (1.85-2.84) |  |
| **Age (years)_** | 40-49 | 1.00 (reference) |  |
|  | 50-59 | 1.13 (0.96-1.31) | *0.1398* |
|  | 60-69 | 0.97 (0.82-1.14) | *0.6811* |
|  | 70-79 | 0.82 (0.66-1.01) | *0.0579* |
|  | 80+ | 0.97 (0.75-1.26) | *0.8263* |
| **Marital Status** | Single | 1.00 (reference) |  |
|  | Divorced/Separated/Widowed | 0.88 (0.74-1.04) | *0.1234* |
|  | Common-law | 0.44 (0.34-0.56) | *<0.0001* |
|  | Married | 0.49 (0.42-0.58) | *<0.0001* |

******* *missing values were added to the reference groups*

**SM Table 3.** Sensitivity analysis* using adjusted measures of association between cancer status and self-reported mental health disorder on the prevalence of life dissatisfaction using logistic model^a^ for OR

| **Mental health**  **disorder Cancer** | **Adjusted^b^ OR (95% CI)** |
| --- | --- |
| No No | 1.00 (reference) |
| No Yes | 2.66 (1.93 - 3.67) |
| Yes No | 5.01 (4.43 – 5.67) |
| Yes Yes | 12.31 (8.31– 18.25) |

*^a^ Weighted to the Canadian population.*

*^b^ Adjusted for sex, age, education level, income, marital status and chronic conditions.*

******* *missing values were added to the reference groups*
